# In the hunt for genomic markers of metabolic resistance to pyrethroids in the mosquito *Aedes aegypti*: An integrated next-generation sequencing approach

**DOI:** 10.1371/journal.pntd.0005526

**Published:** 2017-04-05

**Authors:** Frederic Faucon, Thierry Gaude, Isabelle Dusfour, Vincent Navratil, Vincent Corbel, Waraporn Juntarajumnong, Romain Girod, Rodolphe Poupardin, Frederic Boyer, Stephane Reynaud, Jean-Philippe David

**Affiliations:** 1 Laboratoire d'Ecologie Alpine (LECA), CNRS, UMR 5553, 2233 rue de la piscine Grenoble, France; 2 Université Grenoble—Alpes, France; 3 Environmental and Systems Biology (BEeSy), Université Grenoble Alpes, Grenoble, France; 4 Unité d’Entomologie Médicale, Institut Pasteur de la Guyane, 23 avenue Pasteur, Cayenne, France; 5 Pôle Rhône Alpes de Bioinformatique, Université Lyon 1, France; 6 Institut de Recherche pour le Développement (IRD), Maladies Infectieuses et Vecteurs, Ecologie, Génétique, Evolution et Contrôle (IRD 224-CNRS 5290 UM1-UM2), Montpellier, France; 7 Department of Entomology, Faculty of Agriculture, Kasetsart University, Lat Yao Chatuchak Bangkok, Thailand; 8 Center for Advanced Studies for Agriculture and Food, Kasetsart University Institute for Advanced Studies, Kasetsart University, Bangkok Thailand (CASAF, NRU-KU, Thailand); 9 Institut für Populations genetik, Vetmeduni Vienna, Wien, Austria; University of Washington, UNITED STATES

## Abstract

**Background:**

The capacity of *Aedes* mosquitoes to resist chemical insecticides threatens the control of major arbovirus diseases worldwide. Until alternative control tools are widely deployed, monitoring insecticide resistance levels and identifying resistance mechanisms in field mosquito populations is crucial for implementing appropriate management strategies. Metabolic resistance to pyrethroids is common in *Aedes aegypti* but the monitoring of the dynamics of resistant alleles is impeded by the lack of robust genomic markers.

**Methodology/Principal findings:**

In an attempt to identify the genomic bases of metabolic resistance to deltamethrin, multiple resistant and susceptible populations originating from various continents were compared using both RNA-seq and a targeted DNA-seq approach focused on the upstream regions of detoxification genes. Multiple detoxification enzymes were over transcribed in resistant populations, frequently associated with an increase in their gene copy number. Targeted sequencing identified potential promoter variations associated with their over transcription. Non-synonymous variations affecting detoxification enzymes were also identified in resistant populations.

**Conclusion /Significance:**

This study not only confirmed the role of gene copy number variations as a frequent cause of the over expression of detoxification enzymes associated with insecticide resistance in *Aedes aegypti* but also identified novel genomic resistance markers potentially associated with their cis-regulation and modifications of their protein structure conformation. As for gene transcription data, polymorphism patterns were frequently conserved within regions but differed among continents confirming the selection of different resistance factors worldwide. Overall, this study paves the way of the identification of a comprehensive set of genomic markers for monitoring the spatio-temporal dynamics of the variety of insecticide resistance mechanisms in *Aedes aegypti*.

## Author summary

The ability of mosquitoes to resist insecticides represents a serious threat to the prevention of arbovirus diseases such as dengue, zika and chikungunya. The detection and monitoring of the resistances developed by natural mosquito populations is essential to enabling their management in the field for as long as there are no operating alternatives to the use of insecticides. However, the genetic factors for the biodegradation of insecticides by mosquito detoxification enzymes are still poorly understood prejudicing their early-detection and monitoring in natural populations. Here we used a combination of next-generation sequencing approaches to identify the genetic bases for this resistance mechanism in the mosquito *Aedes aegypti*. Our data confirmed that an increase in the activity of detoxification enzymes in resistant mosquitoes is often triggered by a rise in their gene copy number. We also highlighted mutations likely affecting the regulation and the protein structure of these enzymes in resistant mosquitoes. These findings provide new opportunities to develop simple molecular tests for the early-detection and monitoring of insecticide resistance in the field.

## Introduction

Mosquitoes are considered by the World Health Organization (WHO) as the most dangerous animals in the world, transmitting numerous infectious diseases and causing millions of death worldwide every year. Vector-borne diseases account for 16% of the estimated global burden of communicable diseases. Among these diseases, dengue, chikungunya and zika viruses transmitted by the mosquito *Aedes aegypti* represent a burden in more than 100 countries with over 2.5 billion people at risk [1–3]. Although progresses are being made on vaccine development [4], several limitations remains and vector control by removing larval habitats and using insecticides still remain the first line of defense against these arbovirus diseases [5]. Chemical insecticides are widely used for vector control but the rapid adaptation of *Ae*. *aegypti* to chemical insecticides is now threatening vector control programmes [6, 7]. Resistance affects most insecticides including pyrethroids, the most recent class of insecticides used against mosquitoes [5]. Although alternative vector control tools are being developped [8–12], their global implementation will not be effective until the next decade and managing insecticide resistance represents an important health challenge in the context of the emergence and re-emergence of arbovirus diseases worldwide [13]. With this in mind, characterizing molecular mechanisms underlying insecticide resistance is crucial for tracking down insecticide resistance alleles and improving resistance management strategies [14, 15].

Resistance of mosquitoes to pyrethroid insecticides can be the consequence of various mechanisms, such as non-synonymous mutations affecting the voltage-gated sodium channel targeted by these insecticides (knock down resistance mutations, known as *kdr* mutations), a lower insecticide penetration, its sequestration, and its biodegradation (metabolic resistance) [16, 17]. Knock down resistance mutations and metabolic resistance are known as the two main pyrethroid resistance mechanisms in mosquitoes. While *kdr* mutations are well known in *Ae*. *aegypti* and can easily be tracked using PCR tools [18], molecular mechanisms underlying metabolic resistance are not yet fully understood with a limited set of robust molecular markers [16, 19]. Metabolic resistance is caused by an increased activity of detoxification enzymes such as cytochrome P450 monooxygenases (P450s or *CYPs* for genes), carboxyl/cholinesterases (CCEs), glutathione S-transferases (GSTs) and UDP-glycosyl-transferases (UDPGTs) although other families can be involved [17, 20, 21]. Theoretically, metabolic resistance can be the consequence of an increased expression of one or multiple detoxification enzymes capable of metabolizing the insecticide and/or the selection of variants showing a higher insecticide metabolism rate due to modifications of their protein structure [16]. As over expression is frequently associated with over transcription, most candidate genes have been identified by comparing gene expression profiles between susceptible and resistant populations with expression microarrays. These studies highlighted many candidate genes over-expressed in resistant populations, some of them being later validated as encoding enzymes capable of metabolizing pyrethroids [20, 22]. However, studies performed in different geographical areas often highlighted different candidate genes [20, 22], questionning the universality of genes associated with metabolic resistance. In addition, microarray approaches failed to identify the genomic changes causing the over-production of detoxification enzymes and particular variants associated with increased insecticide metabolism, impairing the identification of DNA markers of metabolic resistance.

Thanks to technical advances, microarrays are now being replaced by next generation sequencing approaches such as RNA-seq and DNA-seq. RNA-seq presents many advantages over microarrays in term of sensitivity and specificity of expression data and allows investigating concomitantly gene expression and polymorphism variations associated with a given phenotype across the whole transcriptome [23, 24]. To date, only few studies used RNA-seq for investigating pyrethroid resistance mechanisms in mosquitoes and most of them compared the expression profiles of a few populations collected from a single geographic area or laboratory-selected strains [25–27]. Recently, Faucon et al. [28] used deep targeted DNA sequencing for identifying genomic changes associated with pyrethroid resistant in multiple *Ae*. *aegypti* populations collected from South America and South-East Asia together with a laboratory-selected strain. This study evidences several gene copy number variations (CNVs) and point mutations affecting detoxification enzymes associated with resistance to the pyrethroid deltamethrin.

In this context, the present study aims at complementing the genomic data generated by Faucon et al. [28] by combining a RNA-seq approach with a targeted DNA-seq approach focused on regions upstream ATG in order to better understand the genomic bases of metabolic resistance to pyrethroids in *Ae*. *aegypti*. The same biological samples as those used in Faucon et al [28] were used in order to combine results obtained from both studies. The importance of CNVs as a cause of over-production of detoxification enzymes was investigated by comparing RNA-seq expression data with targeted DNA-seq data. Polymorphism of upstream regions identified by DNA-seq were cross-linked with RNA-seq expression data and upstream variations associated with the up regulation of detoxification enzymes involved in metabolic resistance were identified. Polymorphism variations identified within detoxification genes were used to identify structural variants potentially involved in increased insecticide metabolism. These results are discussed in regards to the identification of novel genomic markers of pyrethroid resistance in *Ae*. *aegypti*.

## Methods

### Ethics statement

Blood feeding of adult mosquitoes was performed on mice. Mice were maintained in the animal house of the federative structure Environmental and Systems Biology (BEeSy) of Grenoble-Alpes University agreed by the French Ministry of animal welfare (agreement n° B 38 421 10 001) and used in accordance to European Union laws (directive 2010/63/UE). The use of animals for this study was approved by the ethic committee ComEth Grenoble-C2EA-12 mandated by the French Ministry of higher Education and Research (MENESR).

### Mosquito populations

Eight *Ae*. *aegypti* populations of distinct geographical origins were used in the study. These populations were identical (same generation and egg batches) as those used in Faucon et al. 2015 [28] in order to cross link genomic data previously obtained with transcriptomic data generated by the current study. These included three laboratory strains fully susceptible to all insecticides (S populations) and five populations showing elevated resistance to the pyrethroid insecticide deltamethrin (R populations). Field resistant populations were collected in Thailand (NakhR and PhetR) and French Guiana (CaynR and StGeR) during 2014–2015 and maintained in insectaries for 2 generations. In addition to field resistant populations, one laboratory strain selected with deltamethrin for five generations at the adult stage was included in the study (DeltaR population selected from the ‘Bora-Bora’ strain, 80% mortality at each generation). Susceptible strains included the ‘Liverpool’ strain (LivpS) initially colonized from Africa and used for *Ae*. *aegypti* genome sequencing, the ‘Bora-Bora’ strain (BoraS) initially colonized from French Polynesia and the ‘New-Orleans’ strain (NwOrS) initially collected from southern USA.

Previous work showed that the populations used in the current study display a broad range of resistance to deltamethrin with the lethal dose killing 50% of individuals (LD_50_) after one hour exposure varying up to 750 fold between susceptible strains and the most resistant populations from South-America (Table 1 and [28]). Resistant populations from Thailand showed an intermediate resistance level (~ 250 fold) while the laboratory-selected strain DeltaR showed a moderate resistance level (~ 5 fold). The time necessary to knock down 50% of individuals (KDT_50_) varied from 11 min for susceptible populations to more than 8 hours for the most resistant ones. As described in Poupardin et al. 2014 [29], the NakhR population also showed a significant resistance to the organophosphate temephos. Previous genotyping of *kdr* mutations revealed that resistant populations from South-America carried the V1016I and F1534C mutations at high frequencies (V1016I from 77 to 96% and F1534C from 75 to 100%) while the S989P mutation was not detected. Asian populations carried the S989P, 1016G and F1534C mutations at medium to high frequencies (14 to 68%; 21 to 58% and 22 to 75% respectively) [28].

**Table 1:** Deltamethrin resistance levels.

Population	Origin	Type	Resistance	RR_50_ ^a^	KDT_50_ ^b^
LivpS	Benin	Lab strain	susceptible	1	11.5
BoraS	French Polynesia (Bora-Bora)	Lab strain	susceptible	1	12.1
DeltaR	French Polynesia (Bora-Bora)	Lab strain	slightly resistant	~5	14.2
PhetR	Thailand (Phetchaburi)	Field	resistant	~250	98.2
NakhR^c^	Thailand (Nakonsawan)	Field	resistant	~250	92.9
NwOrS	USA (New Orleans)	Lab strain	susceptible	1	11.9
CaynR	French Guiana, Cayenne)	Field	highly resistant	~750	>500
StGeR	French Guiana, St-Georges)	Field	highly resistant	~750	>500

^a^: Resistance ratios (RR_50_) were estimated based on the dose of insecticide used for paper impregnation leading to 50% mortality (±10%) after 1h exposure.

^b^: KDT_50_: Time (min) necessary to knock down 50% of individuals using papers impregnated with 0.05 g/100 mL deltamethrin following WHO insecticide testing recommendations.

^C^: population showing significant cross-resistance to organophosphates. Data extracted from [28].

### Samples preparation

All mosquitoes used in this study consisted in 2–4 days-old non-blood fed *Ae*. *aegypti* females grown in standard laboratory conditions as described in [28]. For RNA sequencing, three pools of 30 females from each populations not exposed to insecticide were collected and stored in RNAlater (Life technologies). RNA extractions were performed on each pool separately using the RNAqueous-4PCR kit (Life technologies) according to manufacturer’s instructions and followed by a DNase treatment to remove genomic DNA contaminants. For the deep targeted sequencing of promoter regions, mosquitoes were collected as described in Faucon et al. 2015 [28] and genomic DNA was extracted from two pools of 65 individuals using the PureGene Core Kit A (Qiagen). The two DNA extracts were then mixed in equal proportion in order to be representative of 130 individuals.

### RNA sequencing

Three RNA-seq libraries per population (three biological replicates) were prepared using the TruSeq Stranded mRNA sample Prep kit (Illumina) and sequenced on an Illumina GAIIx sequencer as 75 bp paired reads by Hybrigenics-Helixio (Clermont-Ferrand, France). Sequenced reads were assigned to each sample (unplexing) and adaptors were removed. Reads quality was checked for each sample using FastQC. Reads were then filtered based on their length, pairing and quality using Trimmomatic [30] with parameters set as follows: Leading 25; Trailing 25; Minlen 60; Slidingwindow 4–25. Only paired reads were kept. Reads were then mapped to *Ae*. *aegypti* genome (AaegL3 assembly) using Tophat2 [31] with the following parameters: don’t report discordant pair alignments; final read mismatches = 3; intron length = 45–300000; use coverage search. Only read pairs mapping at a unique location (mapQ > 50) were retained. Quantification of transcription levels was performed using the Cuffdiff2 module of Cufflinks implemented into a Galaxy pipeline (http://galaxyproject.org) based on Fragment Per Kilobase exon Model (FPKM) values obtained for each gene across all samples.

Transcription ratios between each resistant and each susceptible strain were computed across all biological replicates using Cuff-diff. Genes showing an FC ≥ 3 (in either direction) and a q value ≤ 0.001 between a given resistant population and all three susceptible strains were considered differentially expressed (DE genes).

Polymorphism analysis was performed with Strand NGS V2.2 (Strand Life Sciences). SNPs were called against the reference genome with the following parameters: ignore spill overs at locations around homopolymer stretch greater than 10; ignore reference locations with homopolymer stretch greater than 10; ignore reference locations with coverage below 10; ignore reference locations with variants below 2; confidence score cutoff 50; error rate 0.0010. For each SNP, allele frequencies were estimated based on the number of read supporting each allele. Only substitutions being polymorphic across populations were retained (*i*.*e*. showing > 5% allele frequency variation). SNPs showing an allele frequency variation > 40% (in either direction) between any resistant population and all three susceptible strains were considered associated with resistance (Diff SNPs). Genic effects were computed based on reference genome annotation and non-synonymous differential SNPs were identified (NS Diff SNPs). Then the potential effect of NS Diff SNPs on detoxification enzyme binding sites was investigated. For P450s, variations were considered as potentially impacting substrate specificity if located within one of the six substrate recognition sites [32]. For CCEs, variations were considered as potentially impacting substrate specificity if located within 5 bp of amino-acids forming the active site after alignment with the LcαE7 esterase [33]. For GSTs, variations were considered as potentially impacting substrate specificity if located near any amino-acid forming the G-site and the H-site after alignment with agGSTD11 and agGSTD6 [34]. For UGTs, variations were considered as potentially impacting substrate specificity if located near any amino-acid forming the DBR1 and DBR2 domains after alignment with UDPGT1-10 [35].

### Cross analysis of RNA-seq and DNA-seq data

RNA-seq data obtained from detoxification genes were cross-compared with targeted DNA-seq data previously generated for all detoxification genes from the same biological samples [28] together with novel targeted DNA-seq data covering up to 1kb upstream ATG of all detoxification genes.

First, expression data obtained from RNA-seq were cross-compared with exome sequencing data previously obtained by DNA-seq in order to confirm the role of copy number variations (CNVs) in the over-expression of detoxification enzymes associated with resistance. Log_2_ expression ratios and Log_2_ CNV ratios were correlated across all resistant populations and genes showing a significant positive correlation (Pearson’s r ≥ 0.7 and p ≤ 0.05) were considered up-regulated as a consequence of CNV through gene dosage effect [36].

Because CNV did not fully explain the over-expression of all detoxification genes associated with resistance, their upstream regions were sequenced in order to identify promoter variations potentially associated with their up-regulation. Sequencing was performed by deep targeted sequencing on genomic DNA extracted from 130 mosquitoes as described in [28] except that target genomic regions consisted in 1 Kb regions upstream ATG of all detoxification genes (S1 Table). Target regions were captured using the Agilent SureSelect target enrichment system and sequenced as 75 bp paired reads by Hybrigenics-Helixio (Clermont-Ferrand, France) on an illumina sequencing platform. Sequenced reads were filtered based on their length, pairing and quality using Trimmomatic [30] and mapped to *Ae*. *aegypti* genome (Aaegl3 assembly) using BWA algorithm [37] as previously described in [28]. SNPs were called against reference genome with a minimum coverage of 30 reads per locus and allele frequencies were computed for each population based on the number of read supporting each allele. Allele frequencies of SNPs located upstream detoxification genes over-transcribed in resistant populations were then correlated to their respective transcription levels (as FPKM or Log_2_ FPKM) across all populations. Variations upstream ATG showing a significant correlation (Pearson’s r ≥ 0.7 or ≤-0.7 and p ≤ 0.05) with the transcription level of their respective genes were identified.

Then, the effect of these upstream variations was examined *in silico* by comparing the occurrence of transcription factor binding sites (TFBS) at each locus between the reference and the variant alleles. For each gene, all possible variant haplotypes were compared with the reference haplotype. Transcription factor binding sites were screened with Promo V3.0 (http://alggen.lsi.upc.es/cgi-bin/promo_v3/promo/promoinit.cgi?dirDB=TF_8.3) using Transfac V8.3 database restricted to animal and factors of at least six bp. DNA patterns showing ≤ 5% matrix dissimilarity with known TFBS were retained and compared between reference and variant haplotypes. Differential TFBS were then manually curated and those likely having a major impact on gene regulation were identified based on *i)* their differential occurrence between reference and variant alleles, *ii)* their known enhancer effect on detoxification genes [38–42], *iii)* their expected effect in regards of the positive/negative correlation between the frequency of the variant allele and transcription level and *iv)* their position relative to core promoter elements and the putative transcription starting site. Putative transcription starting sites were manually identified based on the presence of core promoter elements previously identified in arthropods [42, 43].

## Results

### Sequencing and mapping metrics

RNA-seq analysis generated more than 928 million reads with an average of 116 million reads per population across the three biological replicates (Table 2). More than 92% of sequenced reads were of high quality (see methods). Further filtering reads based on their pairing status (only paired reads retained) and their mapping quality (map Q>50, ≤ 3 mismatch, unique mapping location) allowed retaining an average of 65.5 million reads per population (56.5%) for subsequent analyses.

**Table 2:** Sequencing and mapping statistics.

Sequenced reads^a^	BoraS	LivpS	NwOS	DeltaR	NakhR	PhetR	CaynR	StGeR	%
**Sequenced**									
Total	121.86	142.31	83.61	98.66	127.41	114.69	120.58	119.27	100
Mean per replicate	40.62	47.44	27.87	32.89	42.47	38.23	40.19	39.76	
SE	1.16	4.20	3.45	2.72	1.57	3.54	3.75	4.70	
**Passing QC filtering**									
Total	111.85	131.22	76.99	91.23	116.84	105.49	111.19	110.18	92.09
Mean per replicate	37.28	43.74	25.66	30.41	38.95	35.16	37.06	36.73	
SE	0.51	3.44	2.88	2.16	1.86	3.16	3.60	4.51	
**Mapped**									
Total	66.17	82.18	46.99	54.11	71.19	64.81	69.38	69.60	56.49
Mean per replicate	22.06	27.39	15.66	18.04	23.73	21.60	23.13	23.20	
SE		0.63	1.86	1.48	1.28	1.96	1.74	2.45	3.19	

^**a**^: in million paired reads.

### Differentially transcribed genes associated with deltamethrin resistance

**Fig 1:**
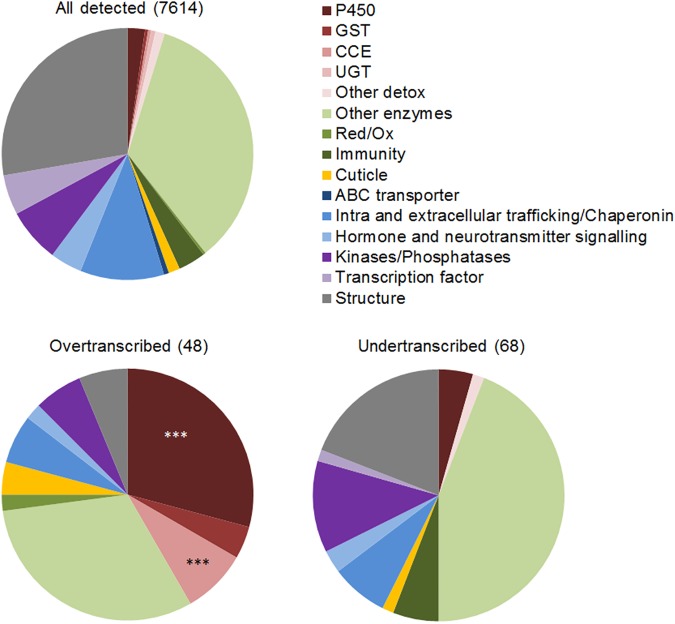
Gene families affected by DE genes in resistant populations. Genes showing a fold change ≥ 3 in either directions, and q-value ≤ 0.001 in any resistant population as compared to all susceptible strains were considered differentially expressed. Biological categories mostly affected were identified by comparing their proportion between all genes detected by RNA-seq and those being differentially expressed using a one-sided Fisher’s exact test (*** p ≤ 0.001).

Differential transcription analysis was performed on the 15491 transcripts showing a transcription level > 0.5 FPKM in each condition pair across all replicates; or > 1.0 FPKM across the two conditions in order to retain genes being silent in one condition but showing a significant transcription level in the other condition. Among these, 7,614 were annotated and manually associated to 15 biological categories (Fig 1 and S2 Table). A total of 118 annotated genes were found differentially transcribed (absolute FC > 3 and q-value < 0.001) in at least one resistant population as compared to all susceptible strains (DE genes). DE genes included 48 genes over-transcribed and 68 genes under-transcribed in at least one resistant population (Fig 1). Comparing the frequency of each biological category between DE genes and all detected genes revealed a strong enrichment of detoxification enzymes belonging to cytochrome P450s (P450s) and carboxyl/cholinesterases (CCEs) among over-transcribed genes. No such enrichment was detected among under-transcribed genes.

**Fig 2:**
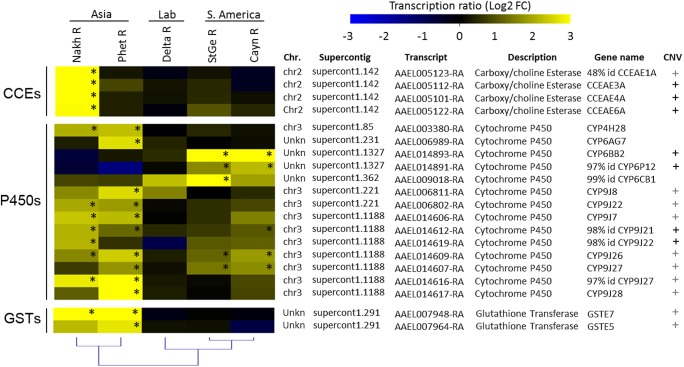
Transcription profiles of detoxification enzymes associated with deltamethrin resistance. Color scale shows the mean Log_2_ Fold Change between resistant populations and all susceptible strains. Stars indicate a fold change q value ≤ 0.001. Black ‘+’ marks indicate a significant positively correlation between Log_2_ transcription ratios and Log_2_ CNVs (Pearson’s r ≥ 0.7 and p ≤ 0.05). Grey ‘+’ marks indicate genes belonging to genomic clusters displaying CNV associated with deltamethrin resistance as reported in [28].

Comparing the transcription profiles of the 20 detoxification genes over-transcribed in resistant populations revealed their frequent conservation within regions while more divergence was observed between continents (Fig 2). Fourteen P450s were found over-transcribed in resistant populations, most of them belonging to the *CYP6* and *CYP9* families. Several *CYP9Js* located on chromosome 3 (supercontigs 1.1188 and 1.221) were over-transcribed in Asian populations with some of them also over-transcribed in South-America. Among CYP6s, the *CYP6CB1-like AAEL009018* was over-expressed in all resistant populations while *CYP6BB2* and the *CYP6P12-like AAEL014891* were specifically over-transcribed in South-America. Finally, *CYP4H28* appeared specifically over-transcribed in Asian population while *CYP6AG7* was specific to the PhetR population. Two P450s slightly over-transcribed in the laboratory-selected strain DeltaR (*CYP9J8* and *CYP6CB1-like AAEL009018*) were also found significantly over-transcribed in field resistant populations. Two epsilon GSTs (*GSTe5* and *GSTe7*) were specifically over-transcribed in Asia while four CCEs located on chromosome 2 (*CCEae3A*, *CCEae4A*, *CCEae6A* and *AAEL005123*) were specifically over-transcribed in the Asian NakhR population showing cross resistance to temephos [29].

### Genomic variations associated with the over transcription of detoxification genes

Comparing the transcription profile of detoxification genes over-transcribed in resistant populations with their copy number variations (CNVs) previously inferred from targeted DNA-seq revealed that most detoxification genes differentially expressed in resistant populations belong to genomic clusters affected by CNV variations associated with resistance [28]. Among them, seven showed a significant positive correlation between their transcription level and their CNV (Fig 2 and S1 Fig). These genes include three CCEs (*CCEae3A*, *CCEae4A* and *CCEae6A*), two CYP9Js (*CYP9J21-like AAEL014612* and *CYP9J22-like AAEL014619*) and two CYP6s (*CYP6BB2* and *CYP6P12-like AAEL014891*). Except CYP4H28 and CYP6AG7, all over-transcribed detoxification genes, were located within gene clusters affected by CNV associated with insecticide resistance as described in Faucon et al. 2015 [28].

**Fig 3:**
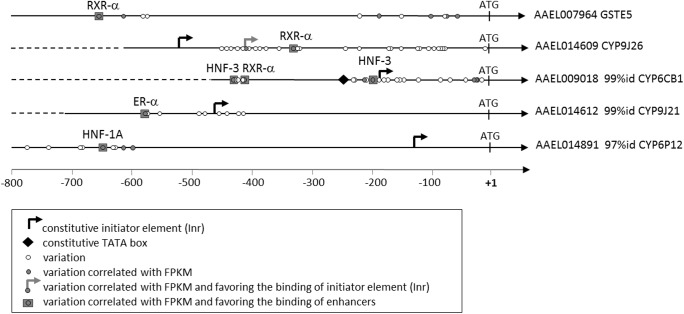
Promoter elements potentially associated with the up regulation of detoxification genes in resistant populations. Only variations correlated with transcription level and favouring the binding of potential enhancer elements are shown. Potential initiation sites (Inr) and TATA boxes are indicated. For each gene, variations detected in the upstream region are shown as empty and filled dots depending on the significance of the correlation between their allele frequency and gene transcription level.

The sequencing of 1 Kb upstream regions of all detoxification genes using targeted DNA-seq identified 188 variations located upstream detoxification genes over-transcribed in resistant populations (S3 Table). Among them, 23 variations showed a significant correlation between their allele frequency and the transcription level of the downstream gene (r ≥ 0.7 and p ≤ 0.05). These 23 variations were located upstream of two GSTs and six P450s (S2 Fig). All of them, except those detected upstream *CYP9J26*, were negatively correlated with transcription levels meaning that the increased frequency of the allele from the reference genome was correlated to the over-transcription of the downstream gene and reciprocally. Seven variations were potentially favoring the binding of transcription factors previously identified as enhancers of detoxification genes in resistant populations (Fig 3 and S4 Table). These included two variations likely to have a major effect on the binding of enhancers: the variation at position -642 (respective to ATG) affecting the gene *CYP6P12-like AAEL014891* favoring the binding of the HNF-1A element and the variation at position -410 affecting the gene *CYP6CB1-like AAEL009018* favoring the binding of the HNF-3 element.

### Non-synonymous variations associated with deltamethrin resistance

**Fig 4:**
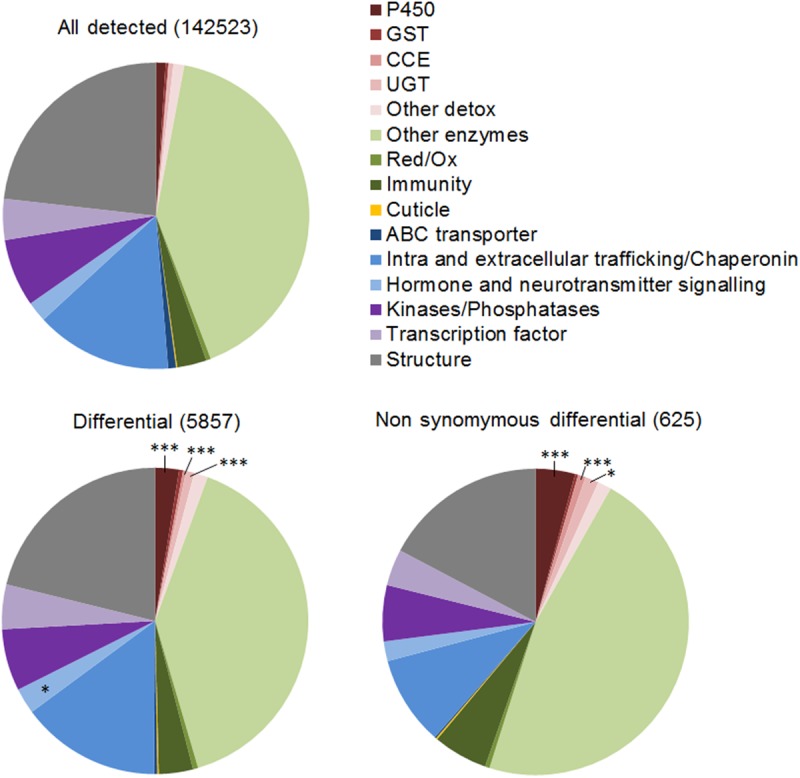
Gene families affected by polymorphism variations in resistant populations. The frequency of each gene family was compared between all detected polymorphic SNPs (top) and both differential SNPs (Diff SNPs) and non-synonymous differential SNPs (NS Diff SNPs) using a one-sided Fisher’s exact test (*p ≤ 0.05,*** p ≤ 0.001).

High quality RNA-seq reads allowed calling a total of 749,596 substitutions against the reference genome, with an average of 300,000 substitutions per population (Table 3). Among them, 244,211 (32.58%) were considered polymorphic (allele frequencies variation > 5% across all populations). Among them, 12,087 were considered associated with resistance (Diff SNPs) as they showed an allele frequency variation > 40% between at least one resistant population and all susceptible strains together with a variation occurring in the same direction in populations from the same region (S5 Table). Nearly half of them (5,857) were located within annotated genes with 625 being non-synonymous (NS Diff SNPs) affecting 436 annotated genes. Comparing the frequency of biological categories between genes where SNPs were called and those affected by Diff SNPs and NS Diff SNPs revealed that biological categories associated with insecticide resistance were significantly more affected (Fig 4). Diff SNPs were significantly enriched among detoxification enzymes such as P450s (2.5 fold), CCEs (9.5 fold) and UGTs (1.9 fold). Although less significant, a slight enrichment was also detected in genes related to hormone signaling and neurotransmitters (1.3 fold). When focusing on NS Diff SNPs, only P450s, CCEs and UGTs remained significantly enriched. Among the transcripts affected by NS Diff SNPs, 41 encode detoxification enzymes, ABC transporters, cuticle proteins and proteins involved in nervous system functioning (S5 Table). Comparing allele frequencies estimated from RNA-seq and from DNA-seq across all detoxification genes revealed a good correlation between both techniques (Pearson’s r > 0.9, S3 Fig).

**Table 3:** Overview of SNP analysis.

	BoraS	LivpS	NwOrS	DeltaR	NakhR	PhetR	CaynR	StGeR	Total	%
Total called	393,506	393,187	189,369	317,695	292,280	328,594	346,445	294,774	749,596	100
Polymorphic substitutions	162,192	148,298	89,652	142,655	108,977	123,696	133,867	127,432	244,211	32.58
Diff SNPs^a^	-	-	-	2,430	6,024	1,348	2,165	2,049	12,087	1.61
NS Diff SNPs^b^	-	-	-	218	576	123	209	187	1,146	0.15

a: Differential SNPs.

b: Non-synonymous differential SNPs.

**Fig 5:**
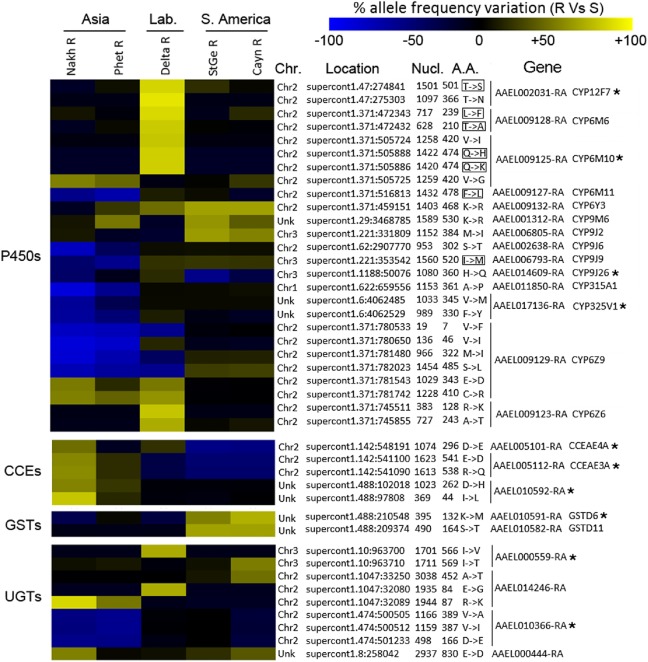
Non-synonymous differential SNPs affecting detoxification genes. Color scale shows the mean allele frequency variation between each resistant strain and all susceptible strains. Yellow/blue colors indicate an increase/decrease frequency of the variant allele in the resistant population respectively. Chromosome, supercontig, base position, nucleotide variation (ref>var), strand, position in cDNA, position in protein, amino-acid change (ref>var), gene accession and gene name are indicated. Stars indicate genes previously affected by NS Diff SNPs as reported in [28]. Boxed amino acid changes indicate variations likely affecting substrate binding site.

Comparing the frequency of NS Diff SNPs affecting detoxification enzymes across populations (Fig 5) revealed that variations associated with deltamethrin resistance were frequently conserved within populations sharing the same genetic background but differed among continents. These NS Diff SNPs impacted fourteen P450s mainly of the *CYP6* and *CYP9* families together with three CCEs, two GSTs and four UGTs. Among these genes, ten were previously identified as impacted by non-synonymous variations associated with deltamethrin resistance based on targeted DNA-seq [28]. These included four P450s (*CYP12F7*, *CYP6M10*, *CYP9J26* and *CYP325V1*), three CCEs (*CCEAE4A*, *CCEAE3A* and *AAEL010592*), one GST (*GSTE6*) and two UGTs (*AAEL000559* and *AAEL010366*). Among all NS Diff SNPs affecting detoxification enzymes, none were located in the potential substrate binding sites of CCEs, GSTs and UGTs while seven were located in P450 substrate recognition sites (SRS). Most of them were detected in the laboratory-selected strain including *CYP12F7* T501S in SRS6, *CYP6M6* T210A in SRS2 and L239F in SRS3 and *CYP6M10* Q414H/K in SRS6 while two were detected in the Asian PheR resistant population (*CYP6M11* F478L in SRS6 and *CYP9J9* I520M in SRS6).

## Discussion

### Detoxification enzymes over expressed in resistant populations

By comparing susceptible and resistant *Ae*. *aegypti* populations from various continents, the present study confirmed the importance of detoxification enzymes in pyrethroid resistance in this species. Indeed, although resistant populations are positive for *kdr* mutations known to confer pyrethroid resistance [44, 45], their transcriptome profiling clearly shows an over-transcription of multiple genes encoding detoxification enzymes previously associated with insecticide resistance [19, 20, 22]. This includes P450s of the CYP9J subfamily from which several members were shown to contribute to deltamethrin metabolism [46]. The gene *CYP6BB2*, strongly over transcribed in South-American populations, was also shown to encode a P450 capable of degrading deltamethrin [45]. Orthologs of *CYP6P12-like AAEL014891* in Anopheles mosquitoes (*i*.*e*. *CYP6P3*, *CYP6P7*, *CYP6P9* and *CYP6M2*) were also shown as encoding enzymes capable of degrading pyrethroid insecticides [47–50]. Recent data obtained by Ishak et al. [51] indicated that the P450 encoded by *Aedes albopictus CYP6P12* degrades pyrethroids. Although the role of *CYP6CB1* in pyrethroid resistance has not yet been functionally validated, this P450 showed an over transcription in multiple resistant populations and was frequently associated with pyrethroid resistance in *Ae*. *aegypti* [20, 52, 53]. Four CCEs were strongly over-transcribed in the Asian resistant population NakhR. However, the over expression of these CCEs is unlikely conferring pyrethroid resistance as this population shows a strong cross resistance to the organophosphate temephos [29] and *CCEAE3A* has been validated as able to degrade and sequester temephos in both *Ae*. *aegypti* and *Ae*. *albopictus* [54, 55]. Finally, *the genes GSTE5* and *GSTE7* were specifically over-transcribed in Asian resistant populations. The over-expression of epsilon GSTs has frequently been associated with insecticide resistance in mosquitoes [56]. The genes *GSTE5* and *GSTE7* are frequently found over transcribed in insecticide resistant populations and *GSTE5* exhibits a DDT dehydrochlorinase activity while the partial silencing of *GSTE7* was associated to an increased susceptibility to deltamethrin [57]. Recent results from Riveron et al. [58] suggest that a specific variant of *Anopheles funestus GSTE2* is involved in permethrin metabolism, supporting the role of epsilon GSTs in pyrethroid resistance. Overall, the present study confirmed the role of particular detoxification enzymes in pyrethroid resistance and identified novel candidates deserving further investigations.

### Genomic variations underlying the over expression of detoxification enzymes

Comparing transcription profiles obtained by RNA-seq and CNV profiles obtained by DNA-seq on the same populations confirmed the importance of CNV as a genomic cause of the over-expression of detoxification enzymes in *Ae*. *aegypti*. The importance of CNV in this species is not surprising as *Ae*. *aegypti* genome is highly infected by transposable elements favouring gene duplication events [59]. Such frequent duplication events affecting clusters of detoxification genes may then rapidly be selected in natural populations by the strong selection pressure caused by chemical insecticides [36].

However, the transcription profiles of detoxification enzymes were not systematically associated with CNV suggesting that the selection of cis- and/or trans-regulatory elements also contributes to the over transcription of particular detoxification genes in resistant populations. The deep sequencing of proximal upstream regions of all detoxification genes using targeted DNA-seq identified 188 variations over transcribed in resistant populations. Among them, only seven variations had their allele frequency correlated with gene transcription levels and were favouring the binding of enhancer elements previously associated with the regulation of detoxification enzymes [38–42]. In insects, mutations in the promoters of insecticide-metabolizing P450s have been associated with their up regulation in insecticide-resistant populations but nuclear factors involved in such process have rarely been characterized [review in 60]. Using luciferase reporter assays, Itokawa et al. [61] recently demonstrated that a mutation affecting the promoter of the duplicated *CYP9M10* further enhances its expression and confers high resistance to permethrin in *Culex quinquefasciatus*. Although our sequencing approach may not cover the full promoter regions of all detoxification genes, it allowed identifying variations upstream two *CYP* genes that may control their up regulation in pyrethroid-resistant populations. Among them, the -410 T>A variation affecting *CYP6CB1-like AAEL009018*, considerably favour the binding of an HNF-3 element. This variation appears particularly interesting as the over-expression of this gene in resistant populations does not appear under the control of CNV and Hepatocyte Nuclear Factors (HNFs) have frequently been associated with the regulation of drug-metabolizing P450s [41, 62, 63]. Secondly, although the transcription level of *CYP6P12-like AAEL014891* is correlated with CNV, the T>A variation at position -642 appears interesting as it considerably favours the binding of an HNF-1A element.

### Point mutations associated with metabolic resistance to deltamethrin

Multiple non-synonymous variations were strongly enriched in resistant populations as compared to susceptible strains. Although a relatively small proportion of them affected detoxification enzymes, statistical analyses revealed that detoxification enzymes such as P450s, CCES and UGTs were significantly more affected than other biological categories confirming the link between detoxome polymorphism and insecticide resistance. As for gene transcription data, polymorphism patterns were frequently conserved within regions but differed among continents confirming the selection of different adaptive trajectories worldwide. Although most variations affecting detoxification enzymes are probably not functionally associated with resistance but rather the consequence of the strong selection pressure undertaken by detoxification genes, some of them may reflect the selection of particular variants showing an increased metabolic activity against insecticides. Such variation has been recently discovered in *An*. *funestus* where a point mutation in the gene *GSTE2* was associated with increased DDT metabolism [58]. Similarly, a conserved mutation affecting the capacity of the esterase αE7 to degrade organophosphates has been identified in various insects [64, 65]. Interestingly other point mutations allowed this esterase to metabolise pyrethroids such as deltamethrin, confirming that the selection of different enzyme variants can confer resistance to multiple insecticides [66–68]. In our study, seven non-synonymous variations were located within the Substrate Recognition Site (SRS) of P450s and may affect the binding and/or the metabolism of insecticides [69]. Five were detected in the laboratory-selected strain DeltaR while only two were detected in field resistant populations and none were shared between populations showing different genetic backgrounds. Whether these mutations are only reflecting the selection imprint affecting these P450s, or are functionally involved in metabolic resistance can’t be ruled out yet. However, if their role in resistance is confirmed, such variability may result from the selection of different adaptive trajectories according to different genetic background and local selection pressures. In such situation, the presence of such mutations in the laboratory-selected strain DeltaR after only a few generations of selection might indicate that, in the absence of major resistance factors, the purification of P450 variants showing an increased insecticide metabolism can occur in the early steps of the selection process. Overall, although some point mutations identified in this study may represent false positives or may result from selection imprints, this data set represent a good basis for investigating the role of the purification of detoxification enzyme variants in insecticide resistance in mosquitoes.

### Novel genomic markers for tracking down metabolic resistance in mosquitoes

Managing insecticide resistance in mosquito vectors represent a significant challenge until efficient alternative control strategies and vaccines will be implemented at a global scale. Such management includes the early detection and tracking of resistance alleles in order to implement adequate resistance breaking strategies in a timely manner.

In vectors of arbovirus diseases, the detection of target-site mutations can be easily achieved through simple PCR assays on individual mosquitoes and allows monitoring the spatio-temporal dynamics of resistant alleles within and among populations. However, such fine monitoring is not yet achievable for metabolic resistance alleles because most validated metabolic resistance markers consists of RNA markers. Indeed, RNA markers are submitted to difficulties and flawless inherent to RNA analysis (sample storage and degradation, high variability, limited biological material, high costs …) impairing the production of individual genotyping data at a high throughput. Recently, Faucon et al. [28] identified particular CNVs as good markers of the over expression of detoxification genes in pyrethroid-resistant *Ae*. *aegypti* populations. However, identifying additional genomic marker associated with the regulation of detoxification enzymes and the selection of particular variants will allow tracking the full spectra of metabolic resistance alleles in the field.

Although the present study identified novel putative genomic markers of deltamethrin resistance, their functional validation is still required which represents a significant research effort. Indeed, though high-throughput sequencing strategies allow studying the molecular bases of insecticide resistance with an unprecedented level of details, improving experimental designs is necessary for narrowing the number of candidate markers to be functionally validated. In addition, reverse genetic approaches such as TALEN and CRISPR/Cas9 appear highly efficient against mosquito detoxification genes [70] and their improvement may greatly accelerate the functional validation of resistance markers in the next few years. In this context, the development of novel molecular resistance diagnostic tools allowing the tracking of the variety of insecticide resistance mechanisms becomes at reach. However, such development will require combining the skills and knowledge of a broad research community through large research networks such as the Worldwide Insecticide resistance Network (WIN) recently developed for vectors of abrovirus diseases [15].

## Supporting information

S1 Table Detoxification genes targeted by DNA-seq analysis of upstream regions. (XLSX)

S2 Table Transcription data of all differentially expressed genes. (XLSX)

S3 Table Variations detected upstream of detoxification genes over-transcribed in resistant populations. (XLSX)

S4 Table Upstream variations associated with the over transcription of detoxification genes in resistant populations. (XLSX)

S5 Table Differential SNPs detected by RNA-seq. (XLSX)

S1 Fig Detoxification genes showing a positive correlation between their expression level and CNV. Correlations were tested for each overexpressed detoxification gene by comparing Log_2_ CNV versus Log_2_ expression ratios. Correlations with Pearson’s r ≥ 0.7 and p ≤ 0.05 were considered significant. CNV data were extracted from [28].
(TIF)

S2 Fig Promoter variations associated with the over-expression of detoxification genes in resistant populations. Only variations located within a 1 kb upstream of detoxification genes overexpressed in resistant populations were considered. Among them, variations showing a significant correlation (Pearson’s r ≥ 0.7 and p ≤ 0.05) between their allele frequency and gene expression level (FPKM or Log_2_ FPKM) across all populations were considered significantly associated with gene expression. For each variation, position on supercontig, nucleotide change (ref>var) and position relative to coding sequence (brackets) are indicated.
(TIF)

S3 Fig Correlation between allele frequencies estimated by RNA-seq and DNA-seq. Only SNPs affecting detoxification genes and detected by both approaches are shown.
(TIF)
