# Supplementary figures and images for: In the hunt for genomic markers of metabolic resistance to pyrethroids in the mosquito Aedes aegypti: An integrated next-generation sequencing approach
Source: PLoS Negl Trop Dis. 2017 Apr 5;11(4):e0005526. doi: 10.1371/journal.pntd.0005526 (PMC5393893; doi:10.1371/journal.pntd.0005526)

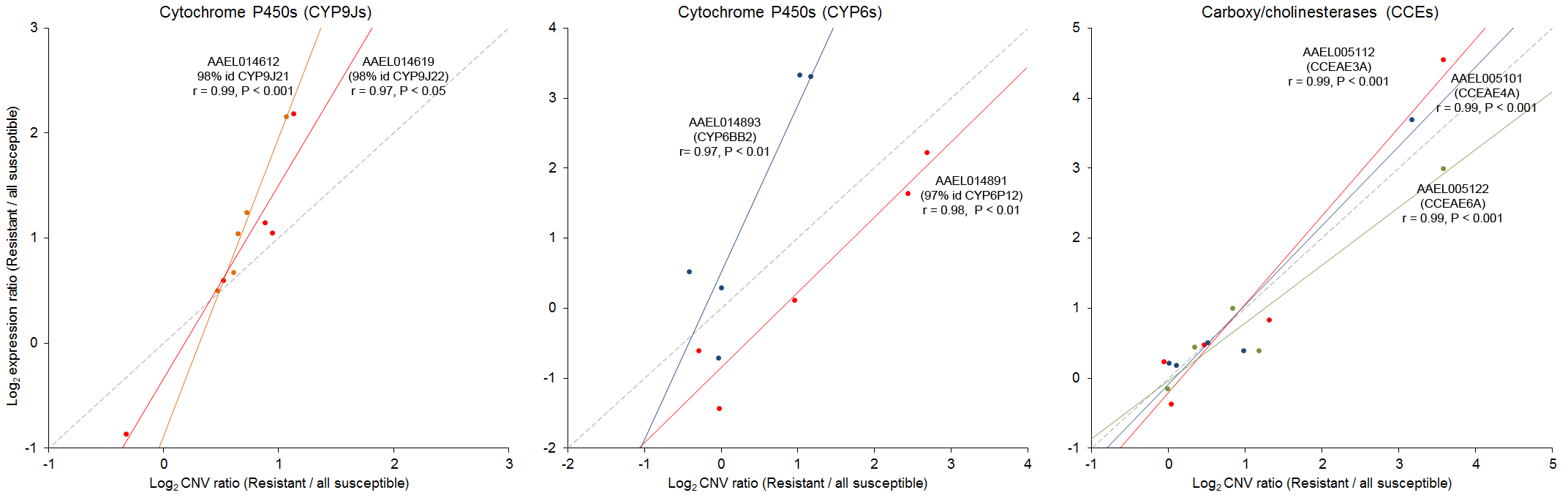

Supplement: S1 Fig — Correlations were tested for each overexpressed detoxification gene by comparing Log2 CNV versus Log2 expression ratios. Correlations with Pearson’s r ≥ 0.7 and p ≤ 0.05 were considered significant. CNV data were extracted from [28]. (TIF) [file pntd.0005526.s006.tif]

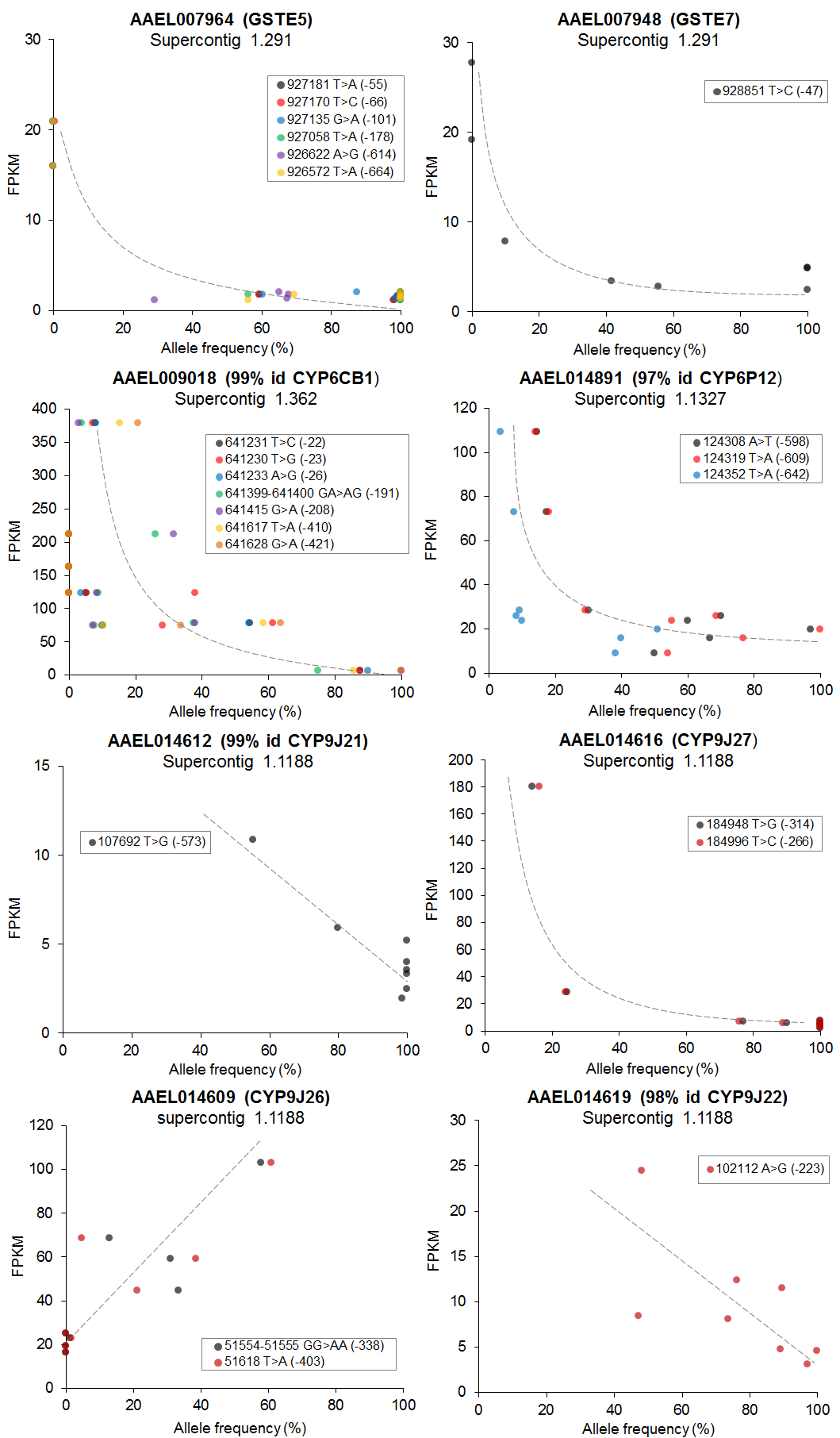

Supplement: S2 Fig — Only variations located within a 1 kb upstream of detoxification genes overexpressed in resistant populations were considered. Among them, variations showing a significant correlation (Pearson’s r ≥ 0.7 and p ≤ 0.05) between their allele frequency and gene expression level (FPKM or Log2 FPKM) across all populations were considered significantly associated with gene expression. For each variation, position on supercontig, nucleotide change (ref>var) and position relative to coding sequence (brackets) are indicated. (TIF) [file pntd.0005526.s007.tif]

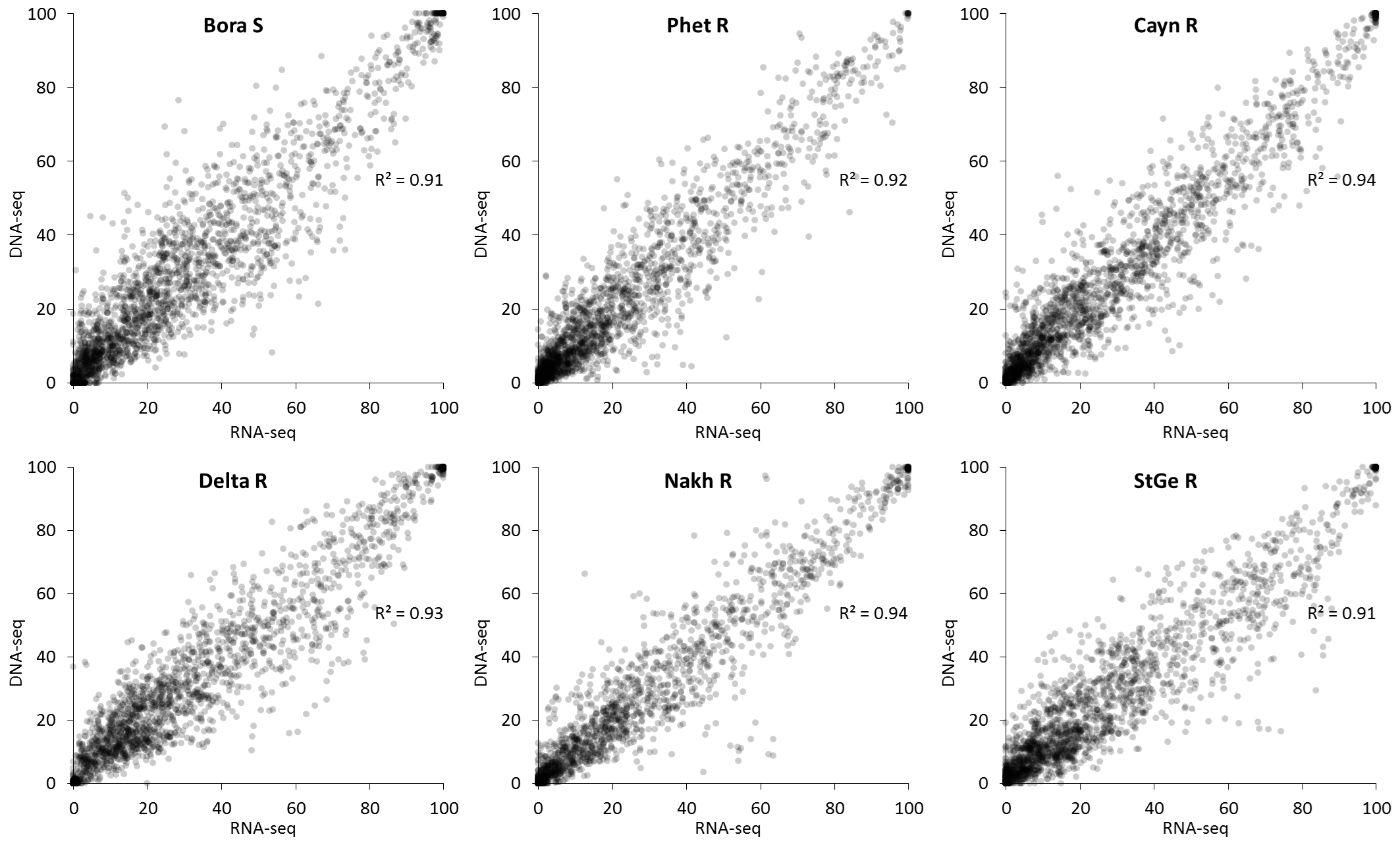

Supplement: S3 Fig — Only SNPs affecting detoxification genes and detected by both approaches are shown. (TIF) [file pntd.0005526.s008.tif]
